# Review: Porous Metal Filters and Membranes for Oil–Water Separation

**DOI:** 10.1186/s11671-018-2693-0

**Published:** 2018-09-12

**Authors:** Huiquan Wang, Xiaoyue Hu, Zunwen Ke, Ce Zhi Du, Lijuan Zheng, Chengyong Wang, Zhishan Yuan

**Affiliations:** 1 Micro-Satellite Research Center, Zhejiang University, Hangzhou, 310027 China; 2 School of Electro-mechanical Engineering, Guangdong University of Technology, Guangzhou, 51000 China; 3 School of Materials and Energy, Guangdong University of Technology, Guangzhou, 51000 China

**Keywords:** Metal pores filter membrane, Wettability, Oil–water separation

## Abstract

In recent years, oil–water separation has been widely researched to reduce the influences of industrial wastewater and offshore oil spills. A filter membrane with special wettability can achieve the separation because of its opposite wettability for water phase and oil phase. In the field of filter membrane with special wettability, porous metal filter membranes have been much investigated because of the associated high efficiency, portability, high plasticity, high thermal stability, and low cost. This article provides an overview of the research progress of the porous metal filter membrane fabrication and discusses the future developments in this field.

## Background

The aquatic environment and health of humans are seriously threatened by offshore oil spills and industrial oily wastewater [1–5]; therefore, many studies have focused on the development of effective oil–water separation methods for pollution control and oil spill recovery. Because of the distinctions in physical properties such as oil phase and water phase densities and conductivities, conventional oil–water separation methods mainly include gravity sedimentation, centrifugation, electrolytic separation, adsorption separation [6], and biodegradation [7]. However, these methods are costly and rather inefficient as they do not prevent oil diffusion.

In recent years, with the progress of interface science and bionics, filter membranes with special wettability have provided a new method for oily wastewater treatment. Metals [8], polymers [9] and fibres [10] with various chemical constituents have been used to fabricate porous and multi-layered membranes. A filter membrane with opposite wettability to aqueous phase and oil phase can form a liquid barrier by preferentially absorbing a certain phase. Based on the equilibrium between the oil–water interfacial tension and the penetrating force from the intercepted liquid phase, the oil can be separated from the water. Compared with the conventional oil–water separation techniques, the use of a filter membrane with special wettability involves easier fabrication and is more efficient and more capable of filtration as well as the recovery of oil phase and aqueous phase from an oil–water mixture.

Because of their low cost, high plasticity, high thermal stability and good mechanical properties, metal materials have been well studied for use as filter membranes with special wettability for oil–water separation. This special wettability can be achieved by coating the membranes with metal nets and a porous metal through physical and chemical methods. In 2004, Feng et al. [11] sprayed hydrophobic polytetrafluoroethylene (PTFE) onto a stainless steel mesh to create a superhydrophobic–superoleophilic filter membrane and reported that this kind of filter membrane was first applied in the oil–water separation field. Subsequently, many porous metal filter membranes with special wettability, fabricated through coating [12], surface oxidation [13] and chemical surface modification [14], were successfully used for oil–water separation. This paper briefly introduces the oil–water separation theory of filter membranes with special wettability and analyses the fabrication, advantages and disadvantages of oil–water separation porous metal filter membranes. The filter membranes are classified into three types according to their properties: filter membranes with superhydrophobic–superolephilic property, with superhydrophilic and underwater superoleophobic properties and with hydrophilic switchable wettability. In addition, future developments in this field are discussed.

## The Principle of Oil–Water Separation

The mechanism of oil–water separation by porous metal filter membranes with special wettability is the superwetting behaviour on the contact interfaces of the solid phase, water phase, and oil phase [15]. Since there is equilibrium between the oil–water interfacial tension and the permeating power from the intercepted liquid phase, this special filter membrane can achieve a selective separation in an oil–water mixture. Therefore, constructing a superwetting surface is the key process to the fabrication of oil–water separation filter membranes.

The surface wettability of the surface material can be characterised by the contact angle, and the main factors influencing the wettability of the surface material are the surface energy and the surface roughness [16–20]. On an ideal smooth surface of solid in air, the contact angle can be expressed by the Young’s equation [21]:

$$ \cos {\theta}_0=\left({\gamma}_{\mathrm{SA}}-{\gamma}_{\mathrm{SW}}\right)/{\gamma}_{\mathrm{WA}} $$

**Fig. 1:**
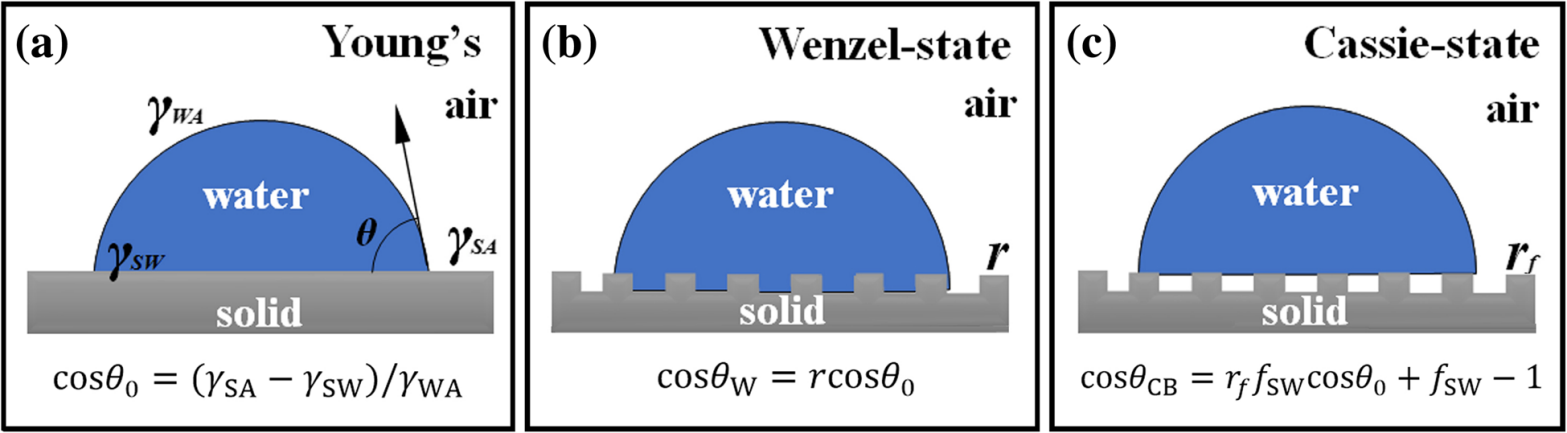
**a** Contact condition of the ideal smooth solid surface in the air and the droplets. **b** Wenzel state [21] when the droplets are in contact with the rough surface. **c** Cassie-Baxter state [22] when the droplets are in contact with the rough surface

In this equation, *γ*_SA_, *γ*_WA_ and *γ*_SW_ represent the interfacial free energy of solid–air interface, liquid–air interface and solid–liquid interface, respectively, which is determined by the chemical constituents of surface material. So the intrinsic affinity of an ideal smooth solid surface to the aqueous phase or oil phase is mainly determined by the surface energy of the solid material, as shown in Fig. 1a.

In 1936 and 1944, Wenzel et al. [22] and Cassie et al. [23], respectively, modified Young’s equation for real surfaces and proposed that the liquid infiltrations on the solid surface at the Wenzel state [22] and Cassie-Baxter states [23] are as shown in Fig. 1b, c. Surface roughness factor *r*, the ratio of the real surface area to its horizontal projection, is introduced into the modified Young’s equation to magnify the affinity of the solid surface to a certain liquid.

Within a measured unit area on a rough surface, there is actually more surface area; therefore, for the same measured unit area, there is a greater intensity of surface energy on a rough surface than a smooth surface [22]. Hence, the surface roughness factor *r* can be regarded as a factor that “magnifies” the affinity of a solid surface to a certain liquid. The superwetting (superhydrophobic or superhydrophilic) surface can be artificially fabricated by building the surface micro/nanostructure to enlarge the intrinsic affinity of the substance to a certain liquid. When the hydrophobic or oleophobic surface is in the Cassie–Baxter state, the air in the micro/nanostructure between the droplet and the solid interfaces causes a low adhesion of liquid to the solid surface, which may result in a surface with self-flowing and self-cleaning functions.

## Oil–Water Separation Filter Membrane Based on Metal Porosity

### Superhydrophobic–Superoleophilic Filter Membrane

The lotus leaf exhibits a superhydrophobic property because of its surface roughness caused by micro/nanoscaled-layered structures and epidermal wax [24, 25]. Inspired by this, the construction of superhydrophobic surfaces has received much attention in recent years, and filter membranes with superhydrophobic–superoleophilic properties have been produced [11, 14, 26–33]. The surface tension of oil phase is usually lower than that of aqueous phase [34, 35]. According to Young’s equation, to create a superhydrophobic–superoleophilic filter membrane surface, the surface energy of the chosen material should be maintained between that of oil (20–30 mN m^−1^) and water (~ 72 mN m^−1^) [36], and the surface energy of the metal used to fabricate the filter membrane should be higher [37] and exhibit hydrophilicity. Therefore, to impart a filter membrane surface with superhydrophobic–superoleophilic properties, the surface energy of the surface in contact with the liquid phase needs to be reduced via coating or chemical surface modification with a micro/nanostructure covering.

#### Coating

This refers to coating a membrane substrate with a complex micro/nanostructure covering by physical or chemical methods. The coating combines the intrinsic hydrophobicity and low surface energy properties of its constituent materials to create an extremely rough micro/nanosurface structure; thus, a filter membrane surface with superhydrophobic and superoleophilic properties is formed on the metal substrates, such as a metal mesh. At present, spray deposition [11, 38, 39], chemical vapour deposition [26], and electrodeposition [40] methods are successfully applied in superhydrophobic–superoleophilic filter membranes fabrication.

**Fig. 2:**
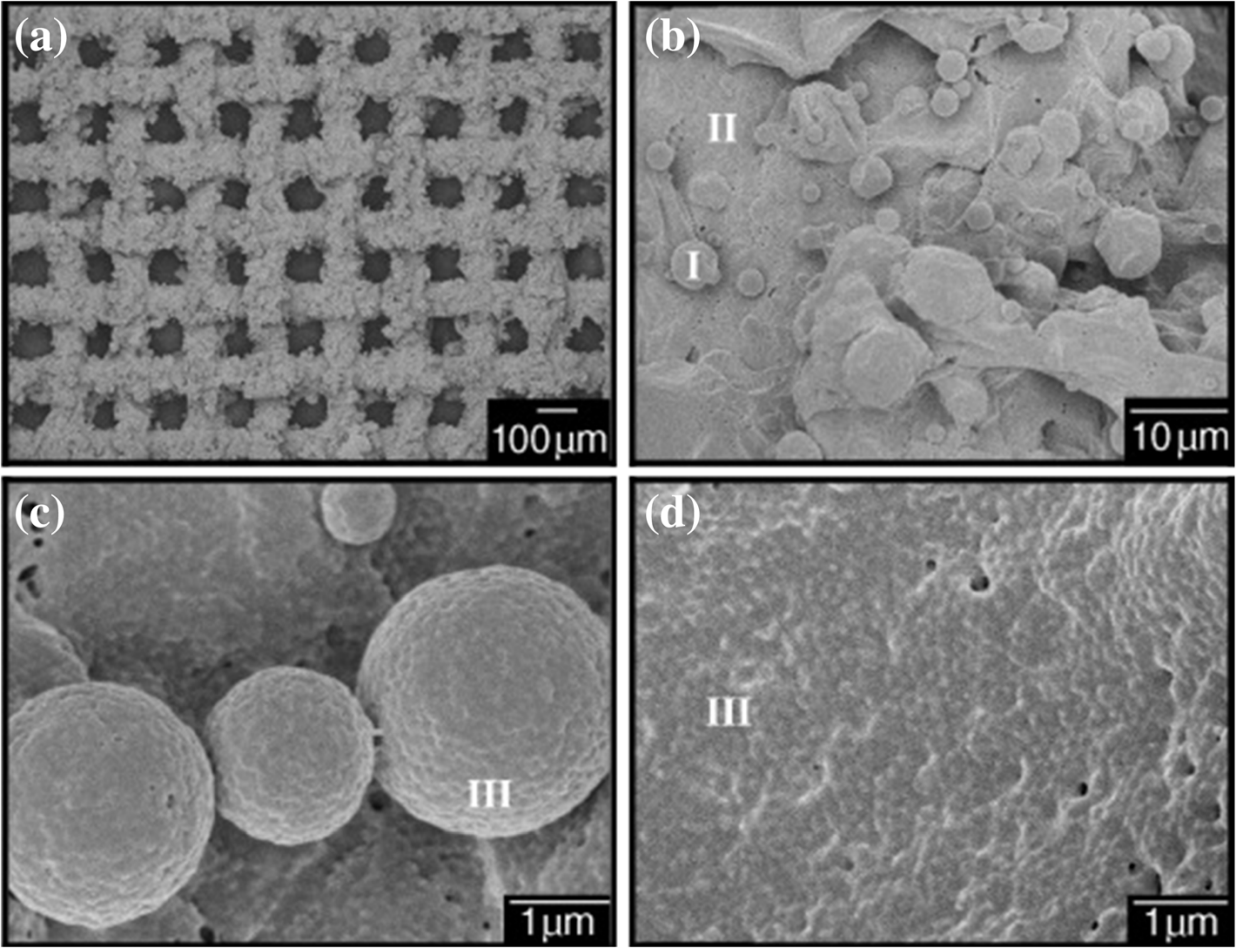
Scanning electron microscopy (SEM) images of the coating mesh film prepared from a stainless steel mesh with an average pore diameter of about 115 μm [11]. **a** Large-area view of the coated membrane [11]. **b**–**d** Enlarged view of the surface microstructure of the coated membrane [11]

In 2004, Feng et al. [11] adopted spray deposition method to deposit a PTFE coating on the surface of a stainless steel mesh to prepare an oil–water separation filter membrane with superhydrophobic and superoleophilic properties, as shown in Fig. 2, and applied this special wetting filter membrane to oil–water separation. The surface of the PTFE coating produced by the above method has microscale spherical protrusions with a nanoscale rough structure, as shown in Fig. 2b–d. This special surface morphology allows the surface of the filter membrane to have large surface roughness, magnifies the intrinsic hydrophobicity of PTFE and imparts superhydrophobic–superoleophilic characteristics to the surface of the filter membrane. Meanwhile, the good stability and chemical resistance of PTFE enable the filter membrane to maintain its surface structure and superhydrophobicity in harsh conditions.

The chemical vapour deposition technique can accurately control the morphology and properties of sedimentary layers by controlling the gas-doping deposition process and has a good application in superhydrophobic surfaces fabrication [26, 41]. Crick et al. (2013) deposited a silicone elastomer on a copper mesh by chemical vapour deposition and produced a porous metal filter membrane with superhydrophobic–superoleophilic properties [26]. This method is easy to operate and has great flexibility, as it only requires the deposition and coating of a superhydrophobic silicone elastomer on the surfaces of complex substrates with different sizes.

Thus far, only few studies have been reported on the influence of temperature on oil–water separation. An increase in temperature results in a decrease in the surface energy of the water droplet, which means a high-temperature liquid wets the surface more easily than a low-temperature liquid [42]. In 2018, Cao et al. [39] developed a copper mesh with a superhydrophobic coating by spray deposition of modified polyurethane and hydrophobic silica nanoparticles. This kind of filter membrane can maintain good hydrophobicity and mechanical stability in a water environment of 100 °C and possesses a bright prospect in industrial application.

#### Chemical Surface Modification

**Fig. 3:**
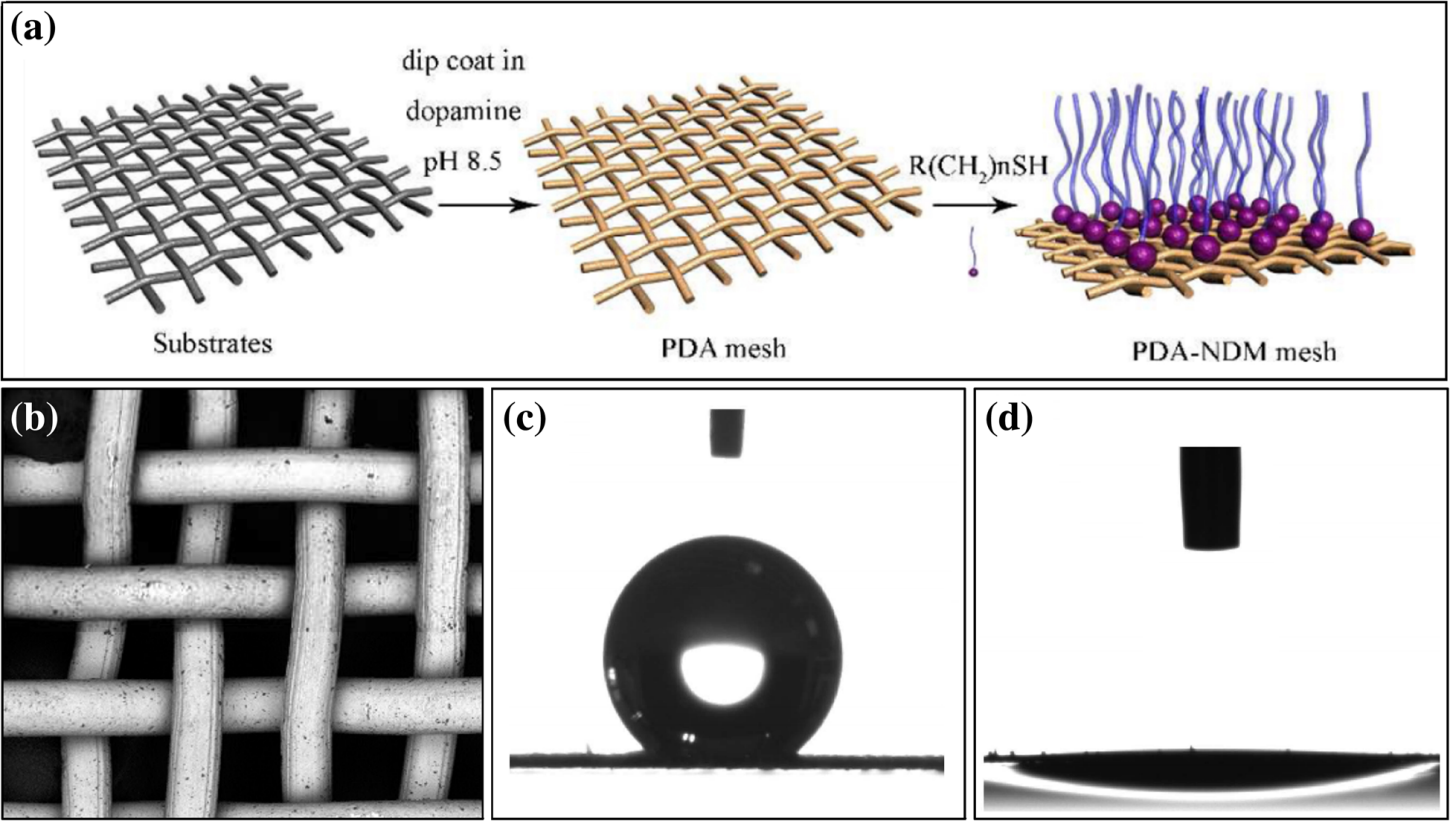
**a** Schematic description of the preparation of polydopamine (PDA) coated stainless steel mesh film and *N*-dodecyl mercaptan (NDM) modified surface through Michael addition reaction [14]. **b** The low-magnification view of the PDA–NDM mesh with an average diameter [14] of approximately 40 μm [14]. **c** The photograph of a water droplet (2 μL) on the PDA–NDM mesh with a contact angle of 143.8 ± 1.0° [14]. **d** A diesel oil droplet (2 μL) spreads and permeates quickly on the mesh [14]

Chemical surface modification involves increasing the surface roughness of the substrate by decorating with a hydrophobic substance, thereby endowing the surface with superhydrophobicity. Inspired by the *Mytilus edulis* foot protein 5 [43–45], Cao et al. [14] conjugated *n*-dodecyl mercaptan (NDM) and a stainless steel mesh membrane coated with adhesive polydopamine (PDA) through Michael addition reaction at ambient temperature, as shown in Fig. 3a, to prepare a superhydrophobic–superoleophilic filter membrane, which successfully achieved oil–water separation. The wettability of PDA–NDM mesh is shown in Fig. 3c, d. The authors introduced a new method to achieve oil–water separation, whereby superhydrophobic–superoleophilic properties are imparted to the surface of a metal filter membrane by decorating the rough surface of the metal substrate with functional groups having hydrophilic and oleophobic properties. By the same principle, Zang et al. [27] modified CuO-grown porous copper mesh surface using perfluorodecyltriethoxysilane; Wang et al. [28] electroplated the Cu nanoparticles on an as-cleaned copper mesh and performed a thiol grafting; Kong et al. [29] deposited cuprous oxide on a copper mesh and realised surface modification using NDM. In all these experiments, a superhydrophobic–superoleophilic surface was constructed and oil–water separation was successfully realised.

Electroplating [29], electrodeposition [32] and chemical etching [33] methods have been used to construct microscale or nanoscale rough structures, but to reduce surface energy, these methods require modifying reagents such as fluorine-containing silanes, alkyl mercaptans and lauric acid, which are harmful to the environment; the modified filter membranes may cause secondary pollution to water. Therefore, chemical surface modification is advantageous as it provides low surface energy following environmentally friendly procedures.

### Superhydrophilic and Underwater Superoleophobic Filter Membrane

A hydrophilic surface has a higher surface energy than an aqueous phase, and thus, it usually exhibits oleophobicity. Inspired by fish scales, Liu et al. [46] developed a superoleophobic and low-adhesive water/solid interface. Water molecules could be trapped in the micro/nanostructures of the underwater superhydrophilic surface because the hydrophilic surface shows underwater oleophobicity. The increasing hydrophilicity of the interface increases the underwater oleophobicity, so that the superhydrophilic surface also possesses underwater superoleophobic property. Considering this phenomenon, various superhydrophilic and underwater superoleophobic filter membranes have been fabricated and applied to oil–water separation.

In superhydrophilic–underwater superoleophobic filter membranes, water is attached to the membrane surface to form an oleophobic liquid barrier, which prevents oil droplets from seeping through, thereby realising oil–water separation [36]. As a result of the underwater oleophobicity and low adhesion to oil, the superhydrophilic material has an excellent underwater antifouling property so that the problem of filter pores being blocked by oil is avoided [47]. However, because of the adhesion of organic pollutants with low surface energy, the superhydrophilicity of this kind of membrane will gradually decrease, which consequently affects the oil–water separation ability.

Metal materials, macromolecule polymers and inorganic non-metallic materials are all applied in the fabrication of superhydrophilic–underwater superoleophobic porous metal filter membranes. The specific methods of fabrication include coating and oxidation.

#### Coating

Coating methods applied to superhydrophilic–underwater superoleophobic filter membrane include spray coating [48–51], dip coating [12, 52], layer-by-layer (LBL) coating [53–55], chemical growth [56] and electrodeposition [57]. Using these methods, the surface of a substrate (usually a stainless steel mesh or copper mesh) is covered with a superhydrophilic–underwater superoleophobic coating.

**Fig. 4:**
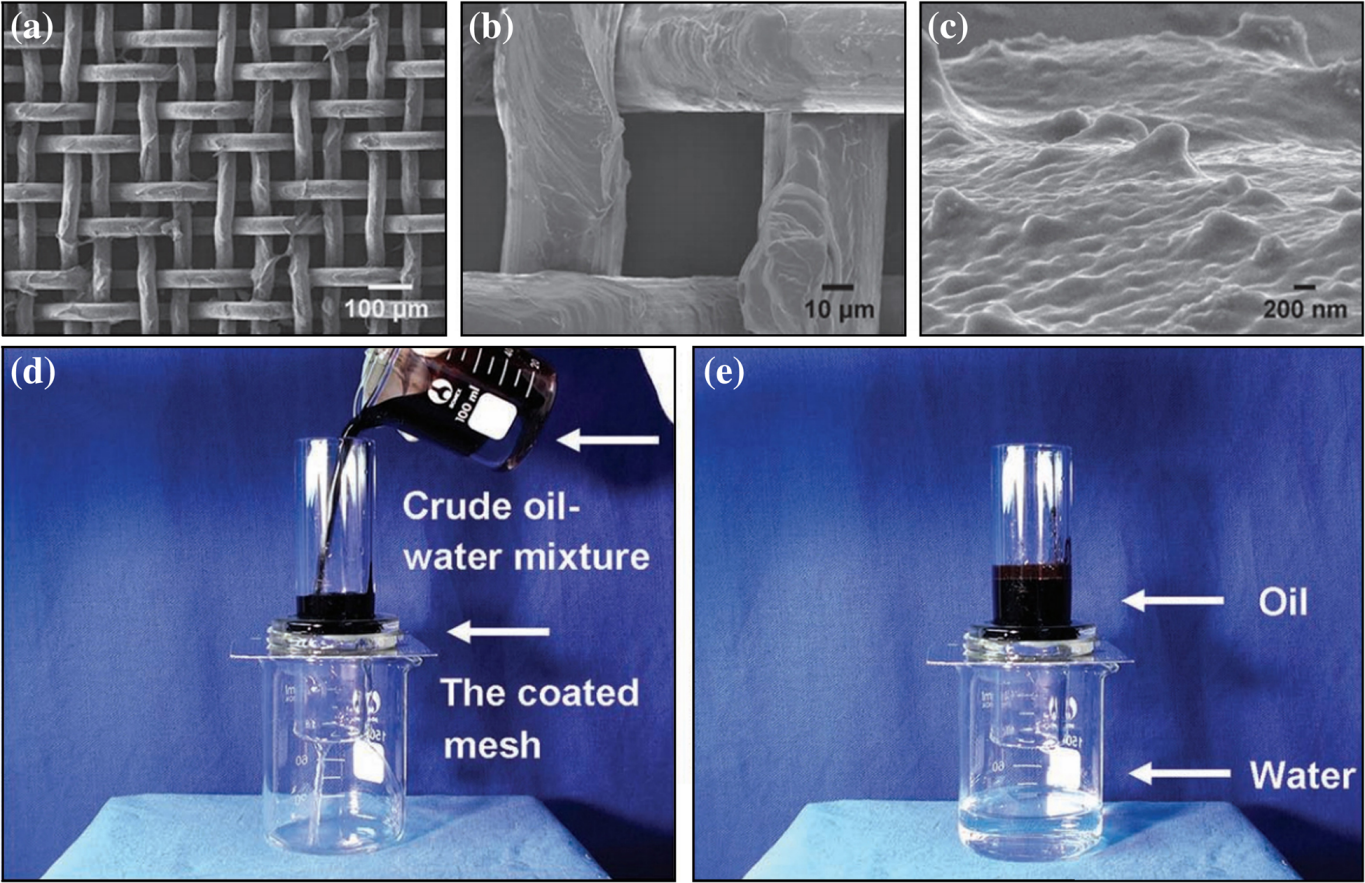
**a**–**c** SEM images of the PAM hydrogel-coated mesh prepared from a stainless steel mesh with an average pore diameter of about 50 μm [12]. **d**, **e** Oil/water separation studies of the PAM hydrogel-coated mesh. The pore size of the mesh is about 50 μm [12]

Hydrogel is widely used in oil–water separation because of its excellent superhydrophilicity and good anti-fouling property [12, 58–63]. Xue et al. [12] first created a superhydrophilic–underwater superoleophobic porous metal filter membrane by coating a stainless steel mesh with polyacrylamide hydrogels as shown in Fig. 4. This filter membrane has a good oleophobic property and is easy to reuse. Moreover, polyacrylamide hydrogel is a fluoride-free and environment-friendly material, and thus, secondary pollution would be avoided during the separation. However, this organic polymer coating is prone to hydration [64], and the polyacrylamide materials degradation requires extreme external conditions. To prepare a self-cleaning oil–water separation filter membrane with underwater low oil adhesion, Dai et al. [62] fabricated a novel guar gum hydrogel-coated stainless steel mesh with both superhydrophilic and underwater superoleophobic properties through an easy and effective dip-coating technique. Natural biodegradable guar gum was used as materials, and the prepared filter membrane exhibited good biocompatibility and easy degradation.

**Fig. 5:**
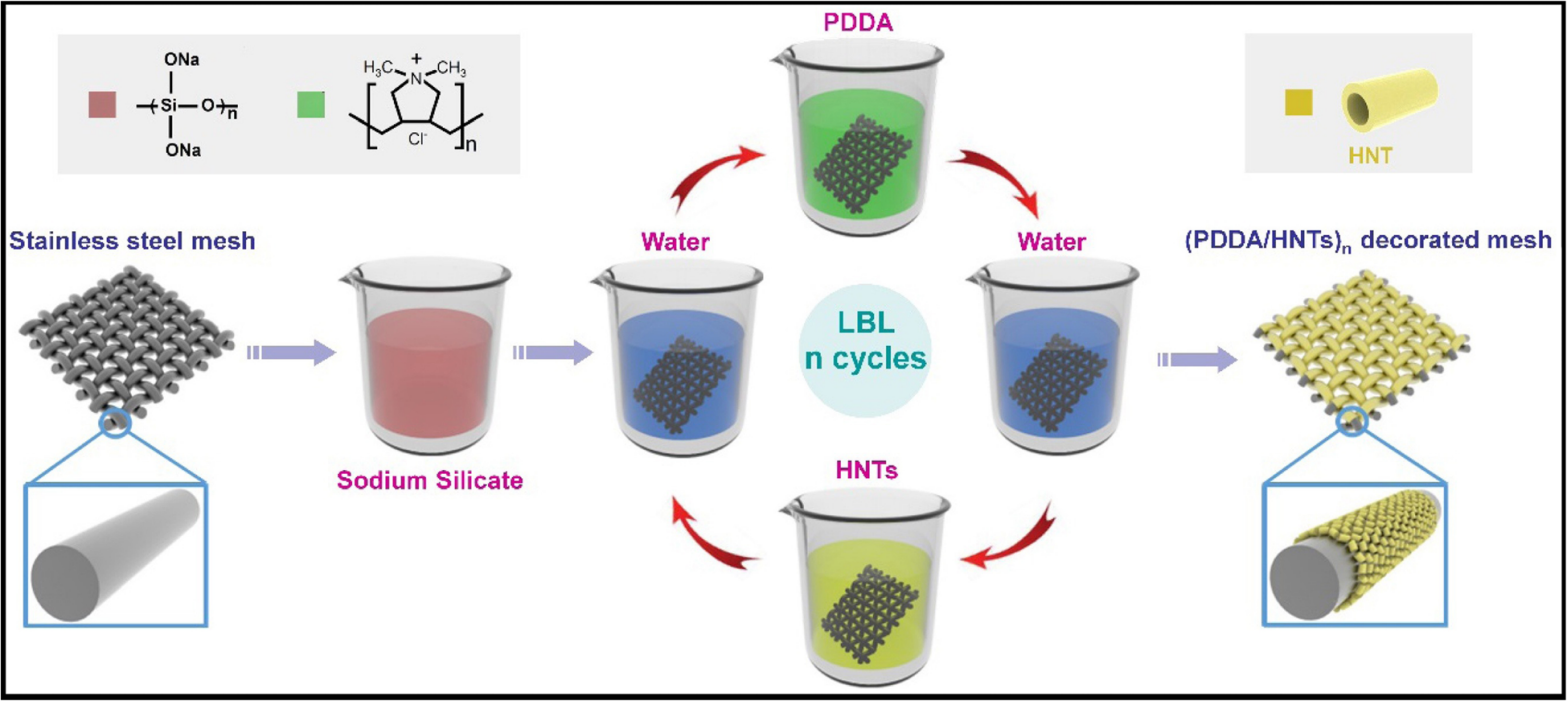
Schematic illustration of LBL assembly process for the fabrication of the (PDDA/HNTs)n decorated mesh [53]

The LBL coating can accurately integrate different functional coatings into a single coating that is largely deposited on the surface of complex structure [54]. Zhang et al. [54] prepared a self-cleaning underwater superoleophobic mesh that can be used for oil–water separation by an LBL assembly of sodium silicate and TiO2 nanoparticles on a stainless steel mesh. Because of the presence of the TiO2 layer, the organic pollutants attached to the filter mesh were catalytically degraded after ultraviolet irradiation. The TiO2 ability to decompose organic pollutants after ultraviolet irradiation has been successfully employed in several studies [8, 49, 54, 65, 66]. Hou et al. (2017) prepared a stainless steel filter membrane with underwater superoleophobicity through an LBL assembly of poly (diallyldimethylammonium chloride) (PDDA) and halloysite nanotubes (HNTs) on a stainless steel mesh [53], as shown in Fig. 5. The stainless steel filter membrane exhibited good chemical and mechanical durability and achieved an oil–water separation rate of over 97%.

#### Oxidation

Oxidation refers to the formation of a metal oxide layer with high surface energy on a metal surface through an oxidation reaction, endowing the filter membrane surface with superhydrophilicity. At present, direct oxidation [13, 65, 67], electrochemical oxidation [47, 55, 66, 68] and laser surface oxidation [8, 69] can be used for the fabrication of superhydrophilic–underwater superoleophobic filter membranes.

**Fig. 6:**
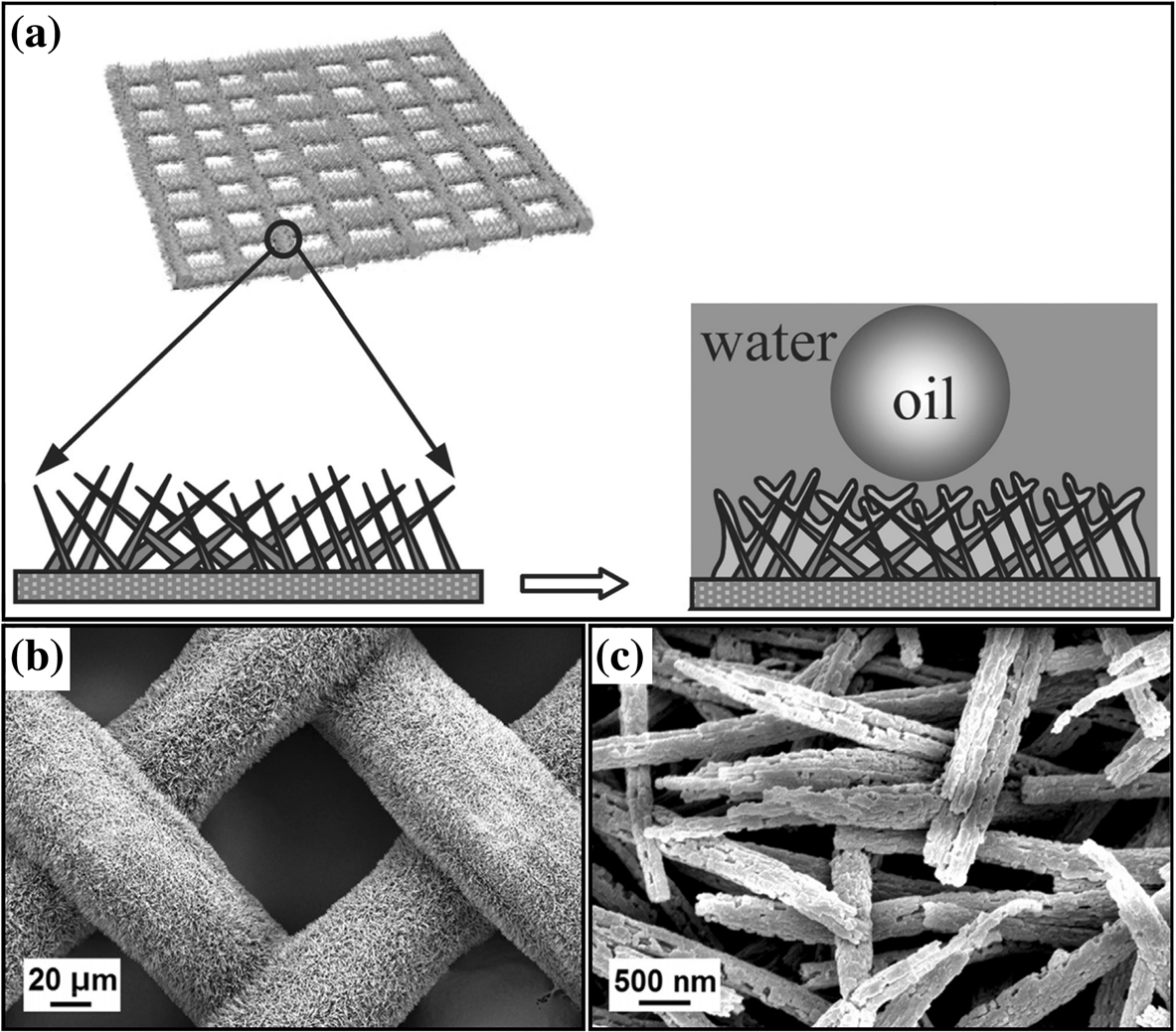
**a** Schematic illustration of oil-wetting on a nanowire-haired membrane with a micro/nano-hierarchical structure in water. [13]. **b**, **c** SEM images of copper mesh coated with CuC2O4 nanoribbons [67]

Feng et al. [13] prepared a nanowire-haired membrane through surface oxidation of a copper mesh in an alkaline aqueous solution with (NH4)2S2O8, and this nanowire-haired membrane with Cu(OH)2 exhibited good superhydrophilic–underwater superoleophobic properties, as shown in Fig. 6a. Compared with the organic filter membrane coating material, this inorganic filter membrane surface has better alkali resistance and antifouling property. However, the Cu(OH)2 nanostructures will be destroyed in the acidic solution and lose their separation ability [67]. Zhuo et al. [67] utilised the above method to prepare a nanowire membrane with Cu(OH)2 and then immersed it in an oxalic acid solution to prepare a nanowire-haired membrane with cupric oxalate, as shown in Fig. 6b, c. This membrane has better acid resistance than the nanostructured membrane with Cu(OH)2.

**Fig. 7:**
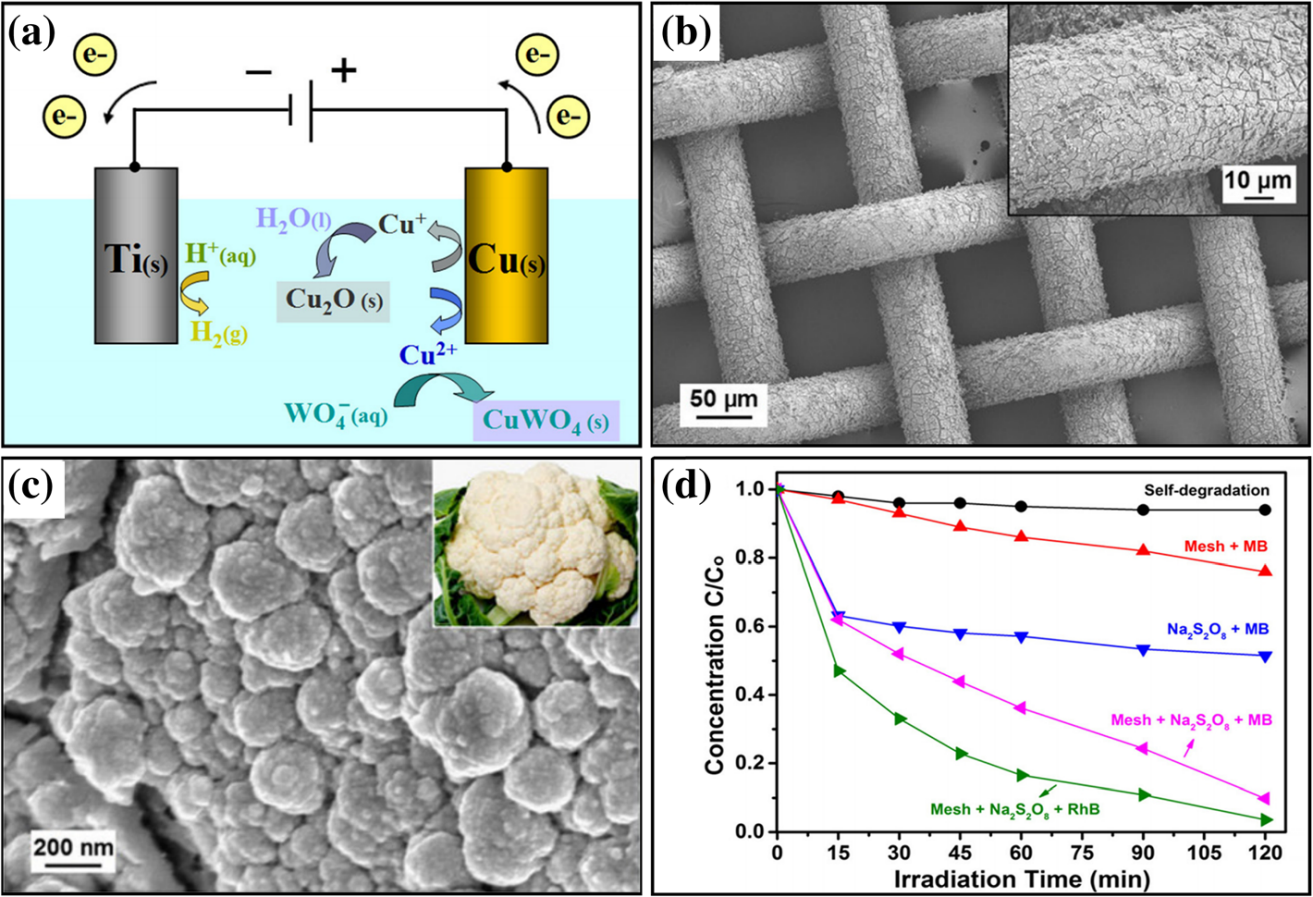
**a** Schematic illustration of the growth of CuWO4@Cu2O on copper substrate by anodisation [68]. **b**, **c** Morphology and structure of the CuWO4@Cu2O film on copper mesh [68]. **d** Photodegradation curves of pollutants in water by using different photocatalysts under visible light irradiation [68]

The direct oxidation method, in which a hydrophilic layer with a special nanostructure is generated through direct oxidisation in a specific solution, has many disadvantages such as the dangers of reagents, harshness of operating conditions and difficulty in controlling the reaction process. In contrast, electrochemical anodic oxidation is an effective alternative to direct oxidation, as it involves a simple operation and low cost, and it can be used to grow ordered nanostructures on a large-area substrate [70]. With this method, the surface morphology and thickness of the oxide layer can be accurately controlled [55] by changing the electrolyte solution, controlling the current density, reaction temperature and time. Through a simple and highly efficient electrochemical anodic oxidation, Pi et al. (2017) prepared a superhydrophilic–underwater superoleophobic Cu_2_S-coated copper mesh [47] with a unique curled plate-like structure, and successfully separated an oil–water mixture. The membrane has low oil adhesion, and unlike the polymer coating, the inorganic coating is stable and does not easily swell in water. Zhuo et al. [68] used electrochemical anodic oxidation, as shown in Fig. 7a, to prepare a CuWO4@Cu2O hydrophilic layer with a hierarchical cauliflower-like structure on a copper substrate, as shown in Fig. 7b, c. This kind of membrane also catalyses the photodegradation of organic pollutants. Different from TiO2, the CuWO4@Cu2O hydrophilic layer can catalyse the degradation of organic pollutants by visible light irradiation, which greatly reduces the difficulty of photocatalytic degradation of pollutants. The photocatalytic degradation of pollutants in water by different photocatalysts is shown in Fig. 7d.

**Fig. 8:**
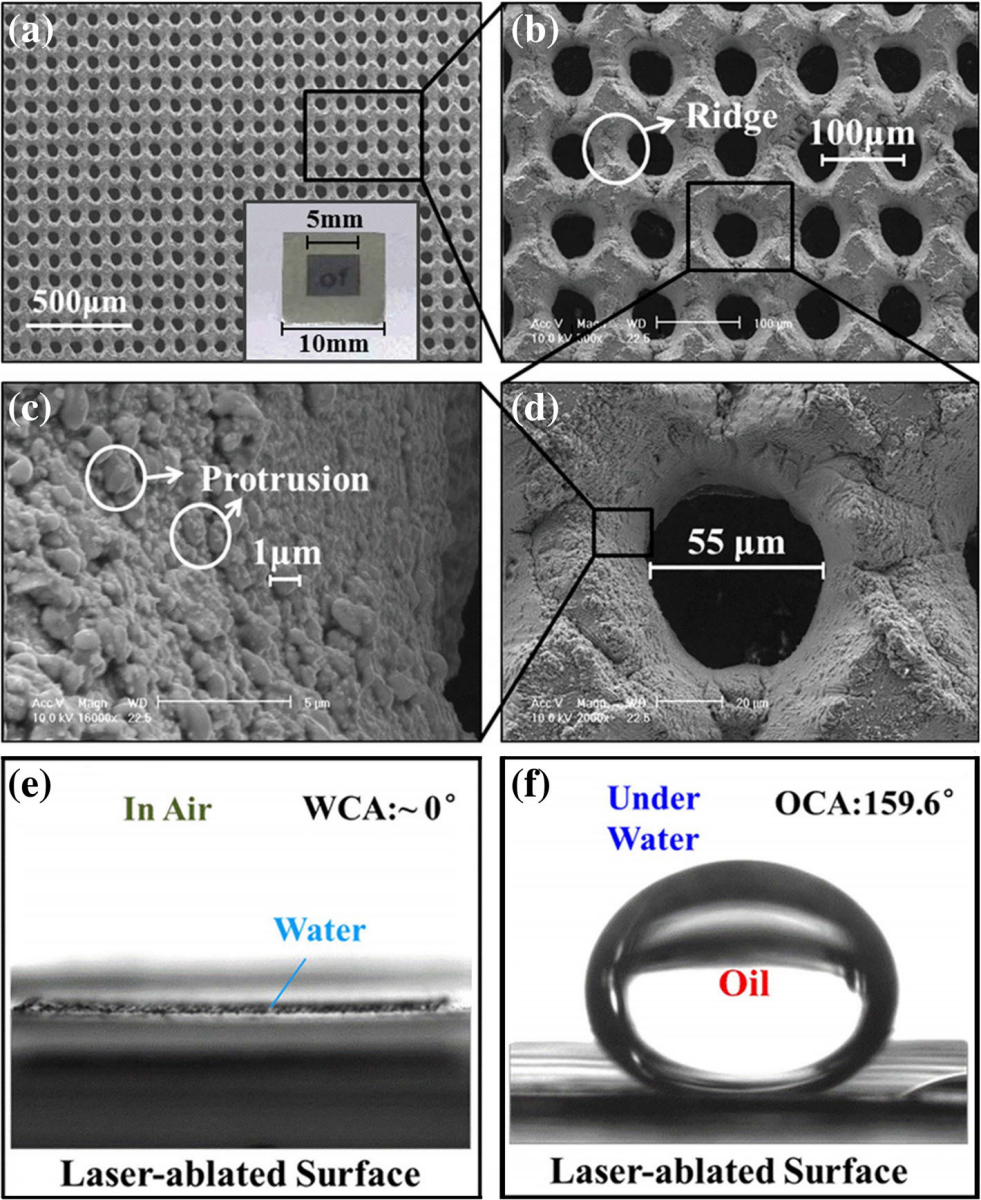
**a**–**d** SEM images of ablated titanium foil fabricated with a laser fluence of 12.4 J/cm2 and a microhole spacing of 100 μm [8]. **e** Wetting behaviour of water droplets on the titanium foil surface after laser drilling [8]. **f** Wetting behaviour of underwater oil droplets on the titanium foil surface after laser drilling [8]

In recent years, sputtering and deposition phenomena in laser processing have attracted widespread attention [71]. Metal surfaces are subjected to laser action, generating high-temperature ablation and plasma. The plasma is deposited on the metal substrate to form an oxide layer with complex micro/nanostructure, endowing the lased metal surface with superhydrophilic property. Ye et al. (2016) fabricated titanium micronpore-array filter membranes using femtosecond laser drilling [8]. As shown in Fig. 8a–d, a TiO2 layer with hydrophilicity was formed on the surface of the membrane by laser processing; the wall of micrometre pores was covered with the microscale protrusions, and ridged protrusions were formed between adjacent pores. These microstructures increased the surface roughness of the filter membrane, which amplified the hydrophilicity of the TiO2 layer on the surface and thus provided the filter membrane with superhydrophilicity and underwater superoleophobicity. The wettability of the titanium foil surface after laser drilling is shown in Fig. 8e, f. Because of the presence of the TiO2 layer with semiconductor property, organic pollutants adhering to the membrane were catalytically degraded after an ultraviolet irradiation.

**Fig. 9:**
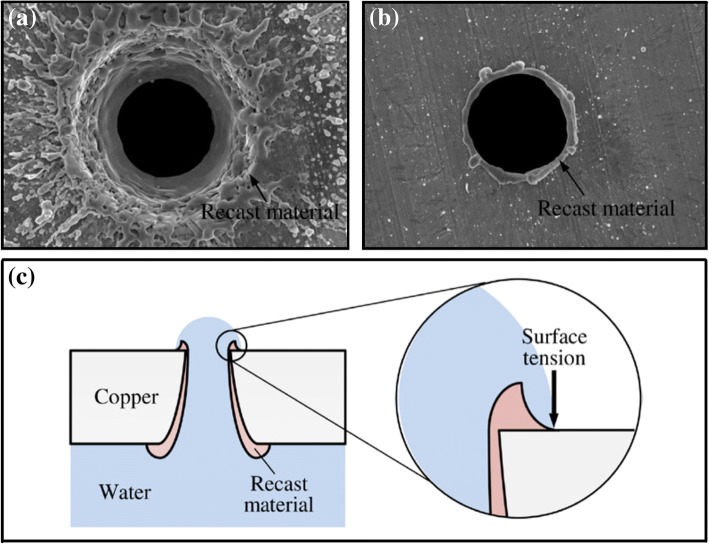
A hole created using laser beam machining. **a** The entrance location. **b** The exit location. (The laser beam conditions are 500 μJ per pulse, 20 kHz and 10 shots) [69]. **c** The location of the water contact line on a hole with recast material at an equilibrium state [69]

Ho et al. [69] fabricated copper micronpore-array filter membranes using femtosecond laser drilling and created a superhydrophilic filter membrane. The entrance location and the exit location of the hole created using laser beam machining are shown in Fig. 9a, b. Because of the surface tension of water and the special annular ridged morphology of the microporous outlet, as shown in Fig. 9c, the water–copper contact line terminates at the microporous outlet. Oil–water separation can be realised based on the different pressures of oil and water passing through micropores arrays. This fabrication method involves the use of chemical reagents for surface modification and is environmentally friendly and simple. However, copper is easily oxidised and corroded by seawater to form materials such as alkaline copper chloride, alkaline copper sulfate and patina [72], which deforms the surface structure of the membrane and affects the oil–water separation ability.

Because of the underwater oleophobicity and low oil adhesion, the superhydrophilic–underwater superoleophobic filter membrane has good underwater anti-fouling performance, and thus, its pores are not blocked by oil [47]. However, because of the adhesion of organic pollutants with low surface energy, the super-hydrophilicity of this membrane will gradually decrease, which will affect the oil–water separation ability. Therefore, methods to fabricate self-cleaning filter membrane surfaces and increase the oil–water separation efficiency and life of filter membranes are challenges that need to be solved in the research field of superhydrophilic–underwater superoleophobic filter membrane.

### Filter Membrane with Switchable Wettability

In the field of oil–water separation, wettability can determine the surface where the controllable conversion of oil filtration or water filtration is realised on a single filter membrane device, and then, an intelligent oil–water separation device can be fabricated, which has good prospects in industrial applications [73].

**Fig. 10:**
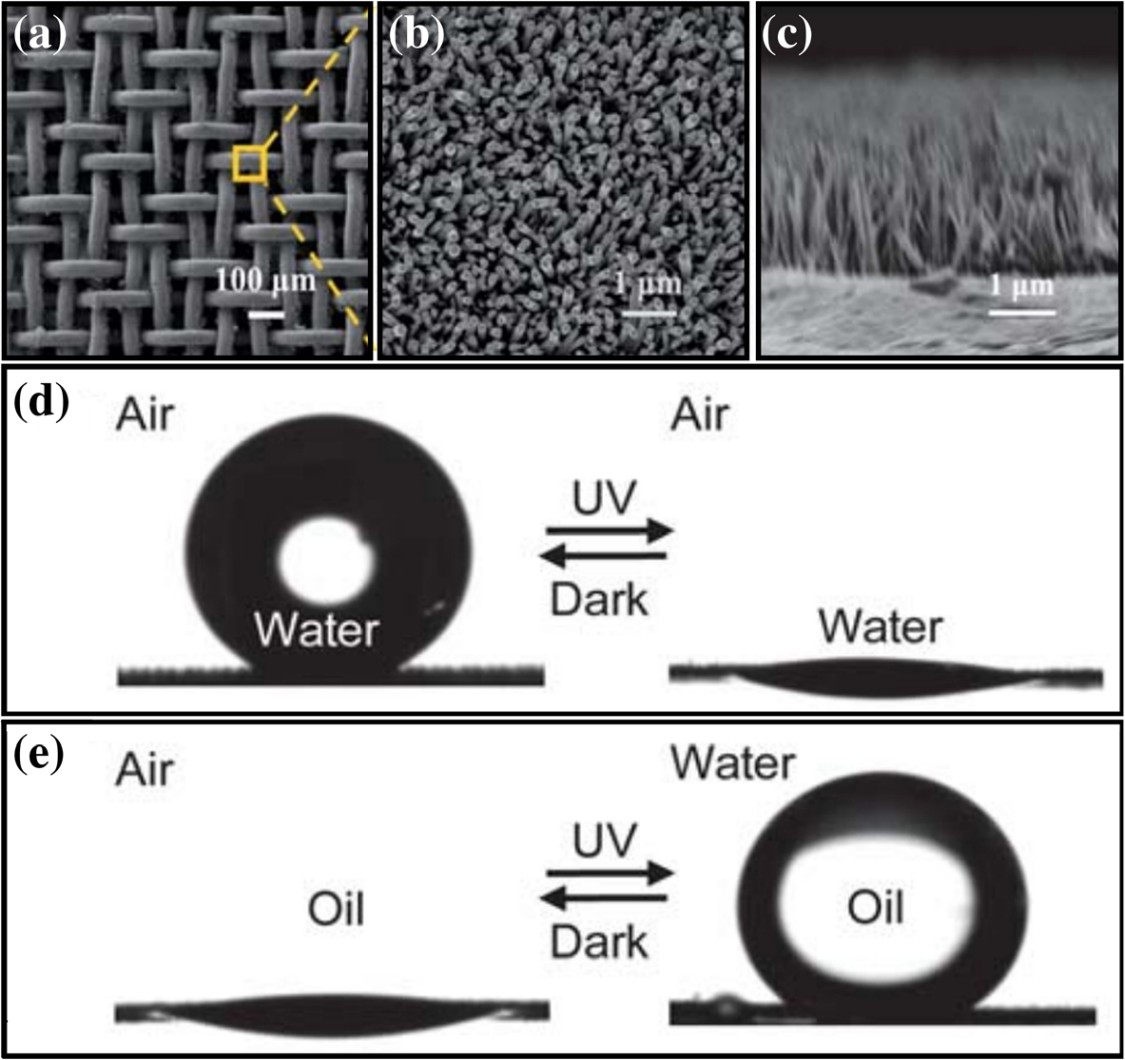
**a**–**c** Schematic diagrams of the SEM images of as-prepared aligned ZnO nanorod array-coated stainless steel mesh films [80]. **d** Photographs of a water droplet on the coated mesh film after dark storage (left) and under UV irradiation (middle) in air with contact angles of ~ 155° and ~ 0°, respectively [80]. **e** Photographs of an oil droplet (1,2-dichloroethane) on the mesh film in air (left) and underwater (middle) with contact angles of ~ 0° and ~ 156°, respectively [80]

Researchers have constructed switchable filter membranes with switchable wettability on textiles [74–77], carbon nanotubes materials [78] and filter paper [79] to achieve an intelligent separation of oil and water. In the studies of a porous metal filter membrane, Tian et al. [80] prepared a ZnO array nanorod-coated stainless steel mesh by a two-step solution approach, as shown in Fig. 10a–c. A photocatalytic oil–water separation filter membrane was obtained. After ultraviolet irradiation, the membrane exhibited superhydrophilic–underwater superoleophobic properties, which prevented the oil in an oil–water mixture from passing through the filter mesh. After being stored in darkness for 7 days or in an oxygen atmosphere for 2 h, the membrane could regain superhydrophobicity–underwater superoleophobicity, as shown in Fig. 10d, e. Yan et al. [81] also used the switchable wettability of a ZnO material in an optical drive to fabricate a photo-induced oil–water separation filter membrane by spraying hydrophobic ZnO nanoparticles and waterborne polyurethane mixtures. With this simple method, the membrane can achieve switchable wettability through alternate ultraviolet irradiation and heat treatment. Yi et al. (2018) developed a thin layer of silver on a copper mesh through a single displacement reaction, and then fabricated a filter membrane with special wettability in the catalytic conversion of ultraviolet rays [82]. The membrane obtained super-hydrophobic properties after heat treatment and superhydrophilic–underwater superoleophobic properties after ultraviolet irradiation.

**Fig. 11:**
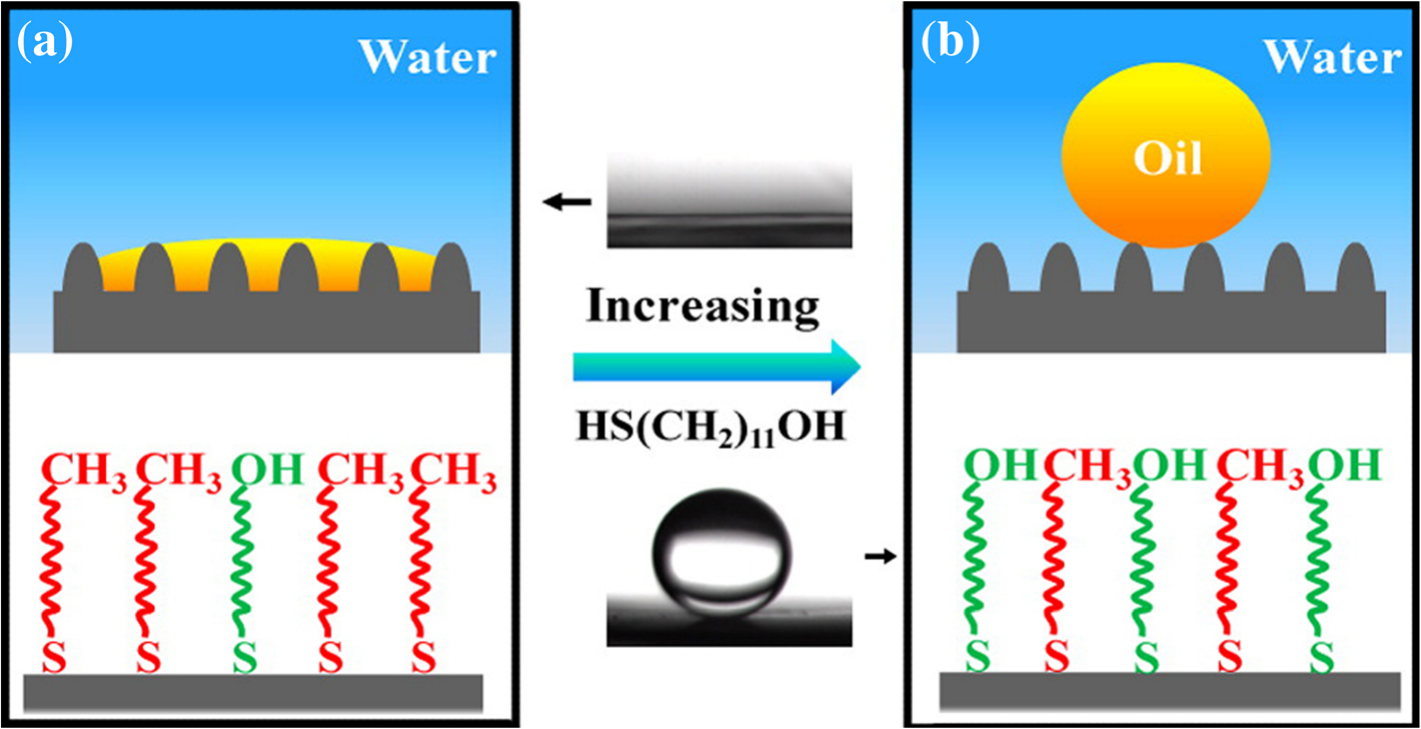
Schematic illustration of underwater oil wettability on the obtained surfaces: for surfaces prepared with XOH ≤ 0.2, the surfaces are mainly covered by the hydrophobic and oleophilic methyl groups ; thus the oil droplet can enter into the microstructures, and the surface would show underwater superoleophilicity (**a**). For the surface prepared with XOH ≥ 0.6, the presence of many hydroxyl groups increases the hydrophilicity of the surface, and water can enter into the microstructures; the oil droplet would reside in the composite Cassie state, and the surface would show superoleophobicity (**b**) [83]

Cheng et al. [83] prepared copper oxides with a micro/nano composite structure on a copper substrate by immersing the copper mesh into a compound solution of (NH_4_)_2_S_2_O_8_ (0.1 M) and NaOH (2.5 M) for 12 h, and then used a mixed mercaptan solution of HS(CH2)9CH3 and HS(CH2)11OH to chemically modify the immersed surface, and finally prepared a water–oil separation filter membrane with controllable surface wettability. When the mole fraction of HS(CH2)11OH in the mixed mercaptan solution approached 0, the surface of the filter membrane exhibited superhydrophobic and superoleophilic properties, as shown in Fig. 11a, and the filter membrane allows only the oil in the oil–water mixture to pass through. When the mole fraction of HS(CH2)11OH was close to 1, the surface of the filter membrane showed superhydrophilic–underwater superoleophobic properties, as shown in Fig. 11b, and the filter membrane allows only the water in the oil–water mixture to pass through. The superhydrophilicity-superoleophobicity of the membrane is due to its surface rough micro-morphology and the hydroxyl and alkyl functional groups introduced into its surface by the mixed mercaptan solution. Hydroxyl exhibits hydrophilicity, while alkyl exhibits hydrophobicity and oleophilicity. Changing the mole fraction of HS(CH2)11OH in the mixed mercaptan solution results in a change in the ratio of the hydroxyl groups to alkyl groups on the membrane surface and consequently alters the affinity of filter membrane surface to water and oil.

**Fig. 12:**
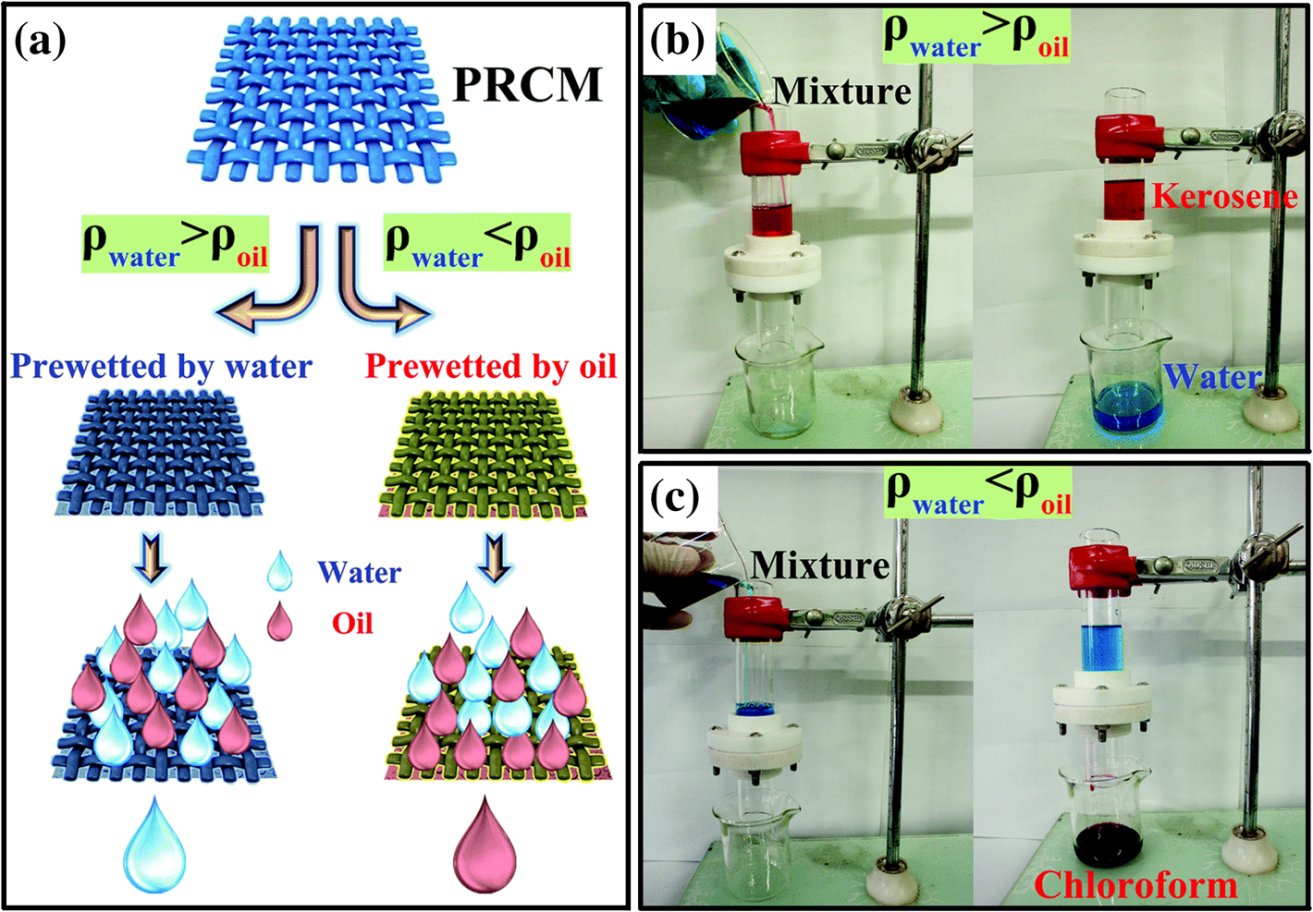
**a** Schematic illustration of the selective separation of oil/water mixtures. PCRM means potato residue coated-mesh. **b** Separation of kerosene–water mixtures (where ρ_wate_r > ρ_oil_). **c** Separation of chloroform–water mixtures (where ρ_water_ < ρ_oil_) (the water was dyed with methylene blue and oil is dyed with Oil Red O to enhance the visual effect) [84]

The pre-wetting of oil–water separation filter membranes exploits the strong affinity of the membrane surface for water and oil to achieve surface hydrophobic and oleophobic conversions as well as an intelligent separation of the oil–water mixture. Li et al. [84] exploited the hydrophilicity of starch, cellulose and pectin in waste potato residue powders and the ability to absorb oil; they sprayed a mixture of waste potato residue and waterborne polyurethane on a stainless steel mesh to fabricate a superoleophobic or superhydrophobic oil–water separation filter membrane catalysed by pre-wetting with water or oil. When the filter membrane is pre-wetted by water, the surface of the membrane acquires underwater super oleophobicity and will allow only water through the filter membrane, as shown in Fig. 12a, b. When the filter membrane is pre-wetted by oil, the surface of the membrane acquires super-hydrophobicity under the oil and would allow only oil to pass through the filter membrane, as shown in Fig. 12a, c.

## Conclusions

In summary, existing oil–water separation porous metal filter membranes can utilise the special wettability of the membrane surface to separate an oil–water mixture and has advantages such as high efficiency, portability, high plasticity, high thermal stability, good mechanical property and low cost. However, there are some aspects where these filter membranes need improvement for effective treatments of industrial wastewater and offshore crude oil spills. First, the environmental adaptability of the filter membranes needs to be enhanced and its working stability in extreme conditions, such as strong acid and alkali, high-concentration salt solution and corrosive liquid, needs to be strengthened, and its mechanical strength should be improved to adapt to the real environment. In addition, the material and modification reagents for fabricating the filter membrane need to be eco-friendly during fabrication and application processes. Furthermore, the fabricating process should be simple, and the manufacturing cost should be reasonable to meet the needs of large-scale production. 3D printing technology has shown outstanding advantages, such as waste minimization, freedom of design, mass customization and the ability to manufacture complex structures [85]. Biomimetic super-hydrophobic structure [86] and superhydrophobic PLA membrane [87] have been printed for oil-water separation. Those results show that 3D printing technology made fabrication process of complex micro-nano structure become easier. Based on this technology, oil-water separation membrane with higher efficient can be gotten in the future. Finally, when the oil–water mixture is in an emulsion state, the filter membrane needs to maintain the oil–water separation capability. An oil–water mixed emulsion is generally defined as oil–water dispersion [88] with a droplet diameter of less than 20 μm, and existing studies of oil–water separation by porous metal filter membranes rarely report the separating conditions for a mixed liquid in an emulsion state. Jiang et al. [52] prepared a superhydrophilic–underwater superoleophobic stainless steel mesh that can preliminarily separate oil–water mixed emulsions using a one-step solution coating method with methyltrimethoxysilane, but this filter membrane cannot completely separate oil–water mixture emulsions, since the apertures of many existing oil–water separation porous metal filter membranes are too large. This remains an urgent challenge in the field of oil–water separation by porous metal filter membranes that need to be solved.

## Abbreviations

HNTs: Halloysite nanotubes
LBL: Layer-by-layer
NDM: *N*-Dodecyl mercaptan
PDA: Polydopamine
PDDA: Poly (diallyldimethylammonium chloride)
PTFE: Polytetrafluoroethylene
